# Will the SAFE Strategy Be Sufficient to Eliminate Trachoma by 2020? Puzzlements and Possible Solutions

**DOI:** 10.1155/2013/648106

**Published:** 2013-05-19

**Authors:** Diane K. Lavett, Van C. Lansingh, Marissa J. Carter, Kristen A. Eckert, Juan C. Silva

**Affiliations:** ^1^Strategic Solutions, Inc., 1143 Salsbury Avenue, Cody, WY 82414, USA; ^2^International Agency for the Prevention of Blindness/VISION 2020 Latin America, 3720 San Simeon Circle, Weston, FL 33331, USA; ^3^Hamilton Eye Institute, University of Memphis, Memphis, TN 38152, USA; ^4^Pan American Health Organization, Bogotá, Colombia

## Abstract

Since the inception of (the Global Elimination of Blinding Trachoma) GET 2020 in 1997 and the implementation of the SAFE strategy a year later, much progress has been made toward lowering the prevalence of trachoma worldwide with elimination of the disease in some countries. However, high recurrence of trichiasis after surgery, difficulty in controlling the reemergence of infection after mass distribution of azithromycin in some communities, the incomplete understanding of environment in relation to the disease, and the difficulty in establishing the prevalence of the disease in low endemic areas are some of the issues still facing completion of the GET 2020 goals. In this narrative review, literature was searched from 1998 to January 2013 in PubMed for original studies and reviews. Reasons for these ongoing problems are discussed, and several suggestions are made as avenues for exploration in relation to improving the SAFE strategy with emphasis on improving surgical quality and management of the mass treatment with antibiotics. In addition, more research needs to be done to better understand the approach to improve sanitation, hygiene, and environment. The main conclusion of this review is that scale-up is needed for all SAFE components, and more research should be generated from communities outside of Africa and Asia.

## 1. Introduction

Trachoma is the leading cause of avoidable infectious visual impairment and blindness worldwide and is caused by the bacteria *Chlamydia trachomatis*, an intracellular obligate parasite [1]. Active infection is characterized by inflammation of the conjunctiva, which leads to subsequent scarring. Repeated infection and scarring leads to an in-turning of the eyelid, or entropion. This causes trichiasis, an extremely painful rubbing of the lashes against the globe, which leads to corneal scarring and blindness. The World Health Organization (WHO) estimates [2] that 21.4 million people have active trachoma and 7.2 million have blinding trichiasis, based on the 2011 provisional country reports from the 53 countries in which trachoma is thought to be endemic. The International Coalition for Trachoma Control roadmap for the elimination of blinding trachoma, 2020 INSight (http://www.trachomacoalition.org/), provides an overview of current data, based on the trachoma atlas (http://www.trachomaatlas.org/), estimating that 110 million people live in confirmed endemic areas and another 210 million in suspected endemic areas in up to 59 countries. However, Brazil and India are excluded due to the lack of an evidence base in these countries. 

**Figure 1:**
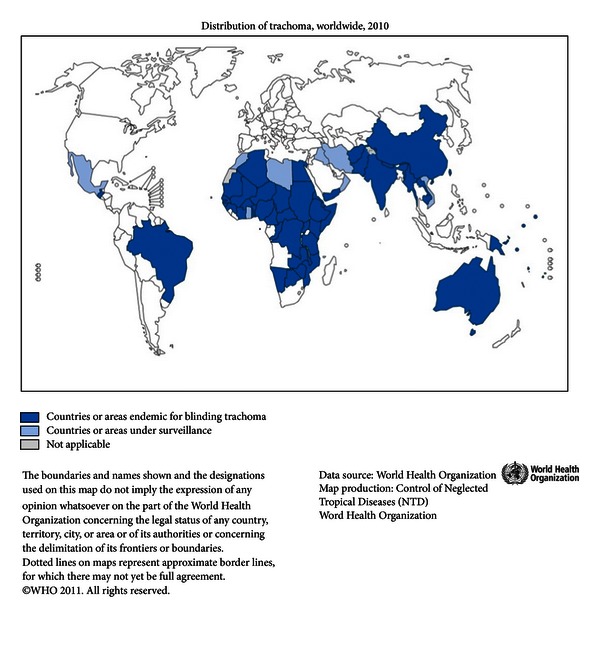
Global map of prevalence of trachoma. Figure courtesy of the World Health Organization [6].

**Figure 2:**
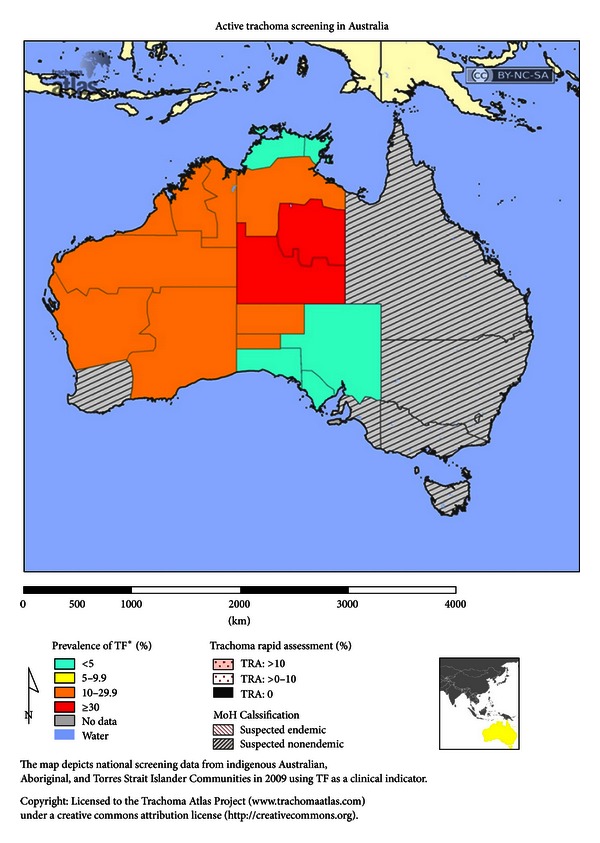
Active trachoma screening in indigenous Australian, Aboriginal, and Torres Strait Islander Communities in 2009 using trachoma follicular as clinical indicator. Figure courtesy of Trachoma Atlas [7].

**Figure 3:**
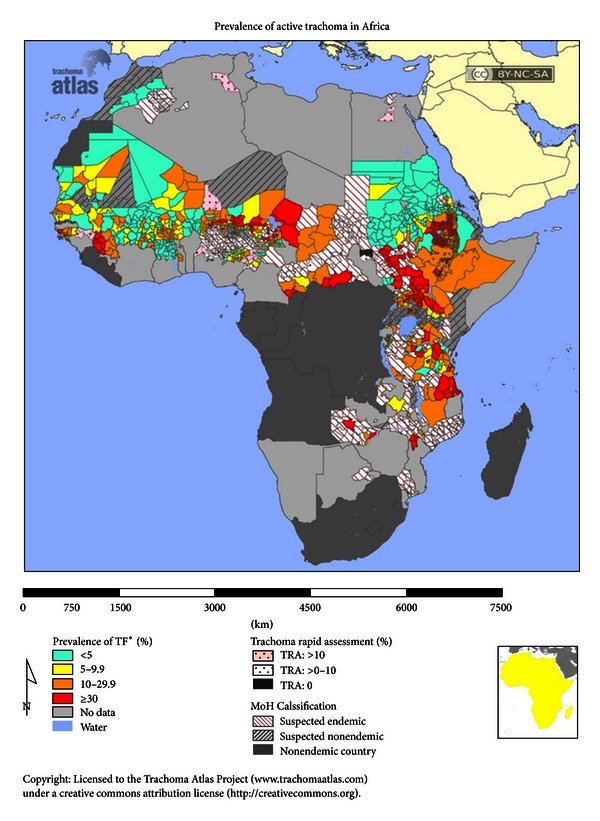
Prevalence of trachoma in Africa using trachoma follicular as clinical indicator. Figure courtesy of Trachoma Atlas [7].

**Figure 4:**
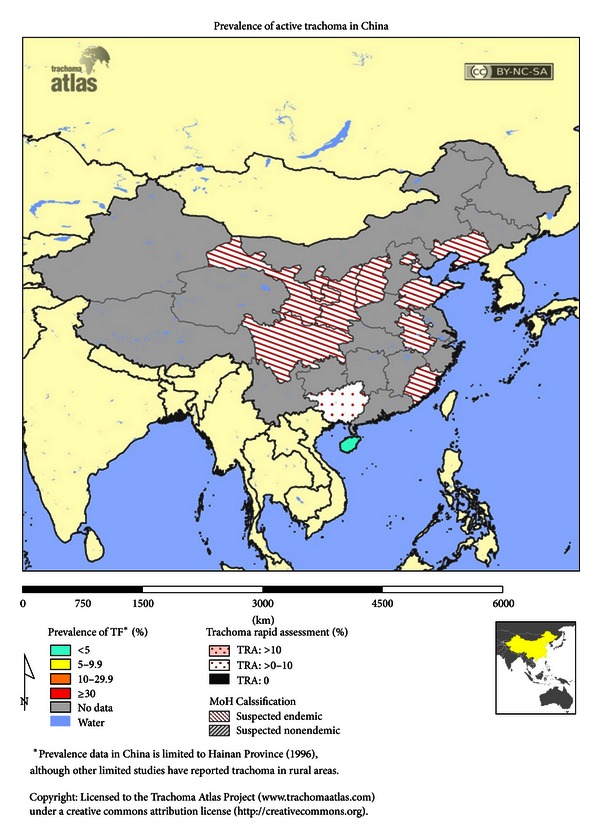
Prevalence of trachoma in China using trachoma follicular as clinical indicator. Figure courtesy of Trachoma Atlas [7].

Endemic trachoma is generally found in undeveloped countries (Figure 1), although Australia has endemic trachoma in its Aboriginal population (Figure 2). Endemic regions include large areas of Africa (Figure 3), the Middle East, Southwestern Asia, regions of India, regions of Southwestern Asia and China (Figure 4), and small regions in South and Central America [3]. Mexico, Morocco, and Oman, which were endemic, have successfully controlled the disease [4]. In many regions, there are hyperendemic endemic areas of trachoma and other areas that have a low frequency of trachoma. Repeated rounds of infection by *C. trachomatis* can occur only where the bacterium is endemic. One attempt [5] at modeling data found that the threshold for scarring of the conjunctiva was 88 repeated infections, and at least 130 infections were required to produce trichiasis. What is important in the findings is the fact that a very high number of infections over a lifetime are needed to reach permanent blindness.

There are only 7 years until the year 2020, the target year for elimination of blinding trachoma worldwide. The goal of this literature review is to evaluate the global strategy to determine whether it is working well and what avenues might be explored to boost its success. 

## 2. Materials and Methods

This literature review is not intended to be exhaustive, but rather, it illustrates the issues with a representative selection of papers, which might engender some bias. Literature was searched from 1998 through February 2013 in PubMed for original studies and reviews that had information on trachoma “prevalence,” “grading” schemes, “infection,” “clinical findings” and correlations between infections and clinical findings, and the SAFE strategy (surgery for “trichiasis,” mass distribution of “azithromycin” and mathematical modeling, and “face washing” and “environmental” issues), using a combination of the term “trachoma” with these quoted phrases or keywords. 

## 3. Clinical Picture

**Figure 5:**
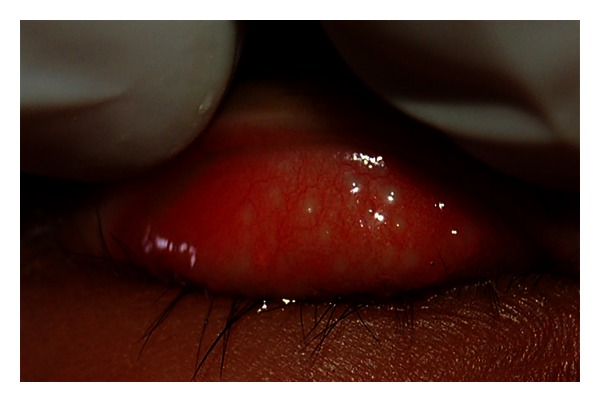
Follicular trachoma (TF stage). Figure courtesy of Hollman Miller, Vaupés, Colombia.

**Figure 6:**
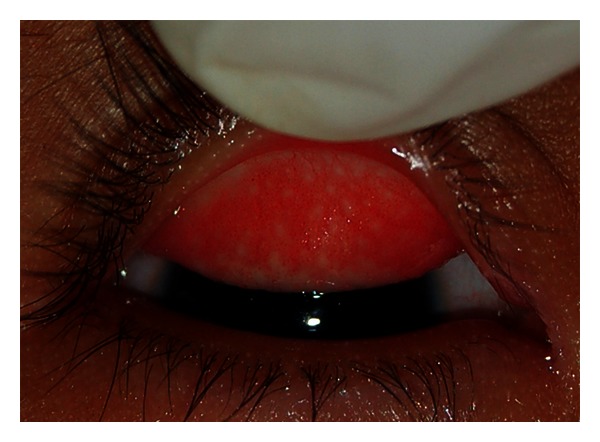
Inflammatory trachoma with Follicular trachoma (TI + TF stage). Figure courtesy of Hollman Miller, Vaupés, Colombia.

**Figure 7:**
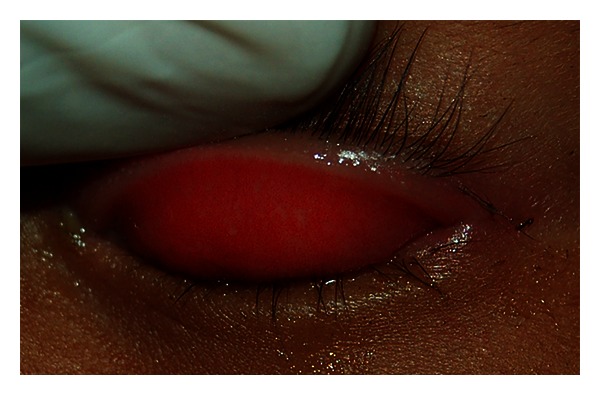
Inflammatory trachoma (TI stage). Figure courtesy of Hollman Miller, Vaupés, Colombia.

The WHO adopted a simplified grading system of the disease (Table 1). Bacterial infection triggers an immune response that results in the formation of macroscopically visible follicles (TF stage, Figure 5). These contain infiltrated cells which release proinflammatory cytokines in the conjunctiva [8]. Elevated IgG and IgA antibodies are in the tears and serum [9, 10]. The conjunctiva become red, swollen (Figure 6), and thickened by the further infiltration of inflammatory cells (TI stage, Figure 7). Limbal follicles may form at the junction of the cornea and sclera, and papillae form from the elevation of the conjunctival epithelium. Ocular pannus, or cornea vascularization, is possible. The presence of follicles constitutes active trachoma, and the active disease process includes the resolution of inflammation as collagenous scar tissue forms. Resolution of the limbal follicles results in “Herbert's pits” depressions on the cornea [11].

**Figure 8:**
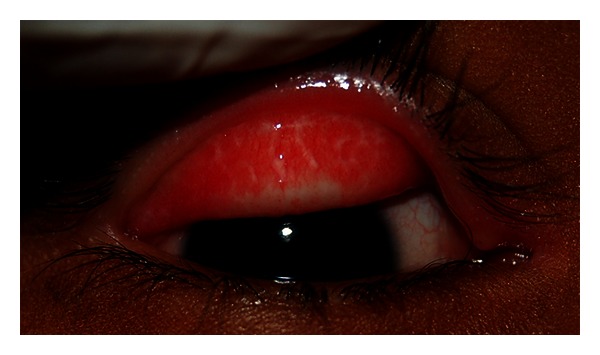
Trachomatous scarring (TS stage). Figure courtesy of Hollman Miller, Vaupés, Colombia.

**Figure 9:**
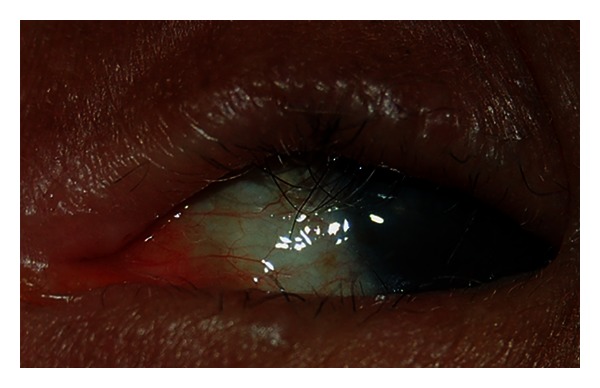
Trichiasis (TT stage). Figure courtesy of Hollman Miller, Vaupés, Colombia.

**Figure 10:**
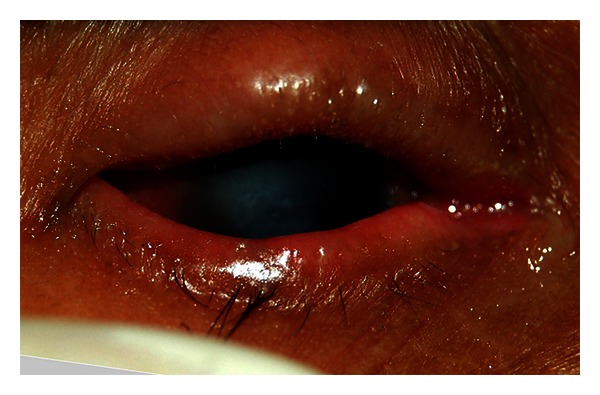
Corneal opacity (CO stage). Figure courtesy of Hollman Miller, Vaupés, Colombia.

Multiple infections over time or intense inflammation lead to further scarring (TS stage, Figure 8). With extensive scarring, entropion, and trichiasis (TT stage, Figure 9), corneal opacity develops (Figure 10), leading to blindness. After repeated and/or severe infections, TT can be detected at around the age of 20, especially in females in highly endemic areas. It is usually detected at around the age of 50. However, in Tanzania, severe infection, repeated infections, and both severe and repeated infection in children under the age of 10 years led to trichiasis within 5 years [12]. While people with untreated trichiasis will ultimately become blind, a study in South Sudan has found that between 5 and 12% of people with trachoma have normal vision with trichiasis [13].

 Children have more infections and more prolonged infection than adults, and the most frequent active infections occur in children with the peak infection rate from 2 to 5 years [14]. They also have a higher bacterial load that leads to more intense infections, supporting data [15] that indicate an increase in antichlamydial cytokines production with age. Thus, adaptive immunity may be achieved with age. However, it is generally understood that female adults, as the primary caregivers of the children, are at higher risk of developing trachoma. A cross-sectional study in 9 hyperendemic villages in Ethiopia found that adult women were 6 times more likely than adult men to be infected with bacteria (the majority with multiple species) and 9 times more likely to be infected with *S. pneumoniae* and *H. influenza* which are commonly found in the nasopharynx of young children [16]. This study further suggests that women are more exposed to bacteria because they are in closer contact with children.

The WHO definition of active trachoma is the presence of TF, TT, or both. Yet, it is difficult to distinguish between disease and detectable infection. *C. trachomatis* causes trachoma, but it cannot be detected in all cases of active disease, may be detected when there are no clinical signs of infection, and a number of other bacteria are often detected with active disease [17–19]. Methods to detect infection include looking at stained cells on slides, cultures of the organism, detection of antigens, and various nucleic acid tests. Gallenga et al. [17] found that polymerase chain reaction (PCR) testing was better in detecting *C. trachomatis* than culturing, immune fluorescent assay, and enzyme-linked fluorescent assay. Yang et al. [19], however, found that tests that amplified ribosomal RNA were much more sensitive, but Keenan et al. [20] suggested that increased sensitivity may still be needed to improve RNA testing. Most of the prevalence rates cited in the literature did not use rRNA testing, so they may be underestimates of the frequency of infection. A study [21] using latent class analysis to detect trachoma was conducted in Ethiopia. (In latent class analysis, observed data is compared with a parameter-optimized latent gold standard, which acts as a composite of all available data to estimate sensitivity and specificity of each test individually. The gold standard applied to most diagnostic tests for trachoma uses a comprehensive clinical scheme rather than the WHO simplified scheme.) The study authors found that follicular trachoma (TF) is a sensitive test (87.3%), intense trachomatous inflammation (TI) is a specific test (88.3%), and PCR is highly specific (100%) but lacks sensitivity (87.5%) [21]. A chlamydial RNA-based gold standard is recommended in lieu of any DNA test after repeated mass azithromycin treatments [22].

Another difficulty in distinguishing between disease and infection is that there is no distinction between active infection and the active disease process. *C. trachomatis* spreads by direct contact with eye, nose, and throat secretions or through contact with towels or other objects that have had direct contact with these secretions. Flies can also transfer the bacterium. There is also the possibility that infection can be spread by aerosol from nasal infection [9]. Andreasen et al. [23] demonstrated differences in genotypes in the same individuals in their nasal and ocular forms of *C. trachomatis*, suggesting that one tissue is not the source of infection for the other tissue, perhaps due to a critical environmental difference between the 2 locations that prevents cross-infection. The same may be true for genital and ocular *C. trachomatis* infections in which different strains or serovars are found at the 2 sites. Serovars A-C cause ocular *C. trachomatis,* serovars D-K cause the genital forms. Serovar typing is based on differences in the major outer membrane protein of the bacterium. Caldwell et al. [24] suggested that the presence or absence of other bacteria is the critical environmental difference.

*C. trachomatis* infection has an incubation period of 5 to 12 days and usually presents as a mild conjunctivitis with a scant watery purulent discharge, but it may be symptomless during some stages. Conjunctival inflammation is “active trachoma,” whether or not the bacterium is present. 

Active infection and active disease are difficult to distinguish due to (1) the short incubation period, during which infection is detectable, but there are no clinical signs; (2) a stage consisting of detectable infection and clinical signs; and (3) a recovery stage in which the infection is not detectable, but there are clinical signs that can last for many months [15, 25]. Active disease is not a good predictor of infection. The simplified WHO grading system is another issue, in which the threshold for active disease is the presence of a minimum of 5 follicles, while an assessment early in infection or a rapid clearance of the bacterium can result in less than 5 follicles. This can lead to infection rates lower than trachoma rates [26]. The WHO guidelines for diagnosing and staging trachoma infections also result in some confusion in the literature, because it is not always clear as to what constituted active infection in a specific report. 

A study [27] that compared the clinical signs of trachoma infection and the infection rate in Aboriginal communities in Australia found that infection, as detected by PCR, was a poor predictor of the presence of clinical disease. Clinical disease was also poorly correlated with infection. They did find, however, that organismal load was strongly correlated with the severity and prevalence of active trachoma by a grading system that allowed finer distinctions than that provided by the simplified system. While the WHO simplified grading scheme has worked well in hyperendemic and moderately endemic areas, it is unlikely to be useful in areas of low prevalence, especially in those areas in which trachoma levels are below 5% [28, 29].

## 4. The WHO “SAFE” Guidelines

In 1997, the WHO founded the Alliance for the Global Elimination of Blinding Trachoma by 2020 (GET 2020). The following year, a World Health Assembly resolution called for trachoma elimination by 2020 using the SAFE strategy of Surgical treatment, Antibiotic treatment for acute infection, Face washing, and Environmental changes to improve sanitation. It was believed that the combined health and development approach would rapidly eliminate blinding (endemic or hyperendemic) trachoma. In a few regions of the world, this has occurred. Blinding trachoma is no longer present in Mexico, Morocco, and Ghana [3, 30]. In most other regions, trachoma has been knocked down only to recur albeit at a much lower prevalence. In Australia, the only developed country where trachoma is endemic, implementation of the entire SAFE program has led to less than complete success [31].

## 5. Surgical Treatment of Trichiasis

There are a number of different surgical procedures that can be used to treat trichiasis [32]. The WHO recommends bilamellar tarsal rotation (BLTR), or lid rotation surgery, for all patients with TT, but it remains unclear if surgery is needed for patients with less severe TT, who tend to epilate the affected lashes and wait until the disease progresses before undertaking the surgery [33, 34]. A newer surgical instrument is the TT clamp. In standard BLTR surgery, a partial thickness incision is made via the skin and orbicularis, followed up with another incision via the conjunctiva and tarsus [35]. The TT clamp uses an integrated eyelid plate and makes one, full thickness incision. While this procedure appears to protect against granuloma formation and some eyelid contour abnormalities, it does not have better surgical outcomes than the standard lid rotation surgery [35].

In Ethiopia, where trachoma is hyperendemic in some regions, trichiasis has a prevalence rate as high as 7% [36]. Thus far, there is no evidence in Ethiopia that trichiasis surgery results in better visual outcomes than epilation in patients with less severe TT, which suggests that epilation may be appropriate for minor TT where surgery is not available or accessible [34]. Nonophthalmologist health personnel have been trained to do trichiasis surgeries as surgeon attrition rates are often high. This occurs because surgeons are also responsible for childbirths, vaccinations, and other disease management, and their time is limited to a few surgeries each year [33]. However, attrition and productivity rates tend to be higher when eye health workers are trained to be surgeons [37]. Interestingly, higher surgical uptake with comparable outcomes occurs in village campaigns, rather than health-center-based surgery [33]. 

A study [36] designed to test the effect of trichiasis surgery on visual acuity found that the WHO—recommended BLTR procedure significantly improved visual acuity. Earlier studies [38, 39] had shown either no improvement or deterioration of visual acuity. However, in the report [38] with no improvement, assessment was at one year after surgery, so the lack of improvement may have been due to recurrence of infection rather than surgical failure. The study [39] with a decrease in visual acuity assessed the subjects from 3 to 4 years after surgery, when recurrence was likely. A recent study [40] in Oman, which is very close to achieving trachoma elimination, found that the rate of blindness decreased significantly in people who had previous lid surgery, but there was no significant difference in blindness and severe visual impairment rates among those with trichiasis and those without trichiasis.

In general, surgical recurrence rates can vary from 7.4% to 62% [33]. High recurrence rates were found in every study [38, 39] for which they have been assessed, but research [41–43] has shown that recurrence may be reduced by a single dose of azithromycin at the time of surgery. No significant additional reduction was achieved by treating additional household members [41]. Given that azithromycin reduces recurrence after surgery, it is possible that there is a continued presence of *C. trachomatis*, that reinfection is occurring, other pathogens are contributing to the process, or that inflammation continues in the absence of reinfection in some people. 

Poor surgical outcomes have been a barrier to the success of some trachoma programs. In the study [35] that evaluated the efficacy of the TT clamp versus standard BLTR surgery, the rates of at least one unfavorable surgical outcome were 60.9% and 63.0%, respectively (adjusted odds ratio (OR) = 0.88, 95%, confidence interval (CI) 0.66–1.18). These high rates are simply unacceptable and further demonstrate the need to improve TT surgical outcomes [35]. In an attempt to define the role of inflammation in surgical failure, Burton et al. [44] studied the proinflammatory cytokine genes at one and 4 years after surgery in a population with 52% recurrent trichiasis, 39.7% of which occurred in the first year. They concluded that IL-1*β* is a key proinflammatory mediator involved in promoting chronic infection. In addition, IL-1*β* is involved in the activation of tissue factors, such as matrix metalloproteinases, that lead to tissue remodeling through their proteolytic activities on collagen. Alteration of collagen may favor increased scarring and contribute to recurrent trichiasis. Bacterial infection was assessed at one year following surgery, and a number of species were found in addition to *C. trachomatis*, which was present in only 3 of 239 samples. One year after surgery, expression of the tissue necrosis factor gene (*TNF*) only increased with conjunctival inflammation and the presence of bacterial infection, whether *C. trachomatis* or one of a number of other organisms. *TNF* expression varied, due to differing alleles of the *TNF* gene, between the 4 ethnic groups studied. A high level of *TNF* expression was also linked to an increased risk of scarring complications [45, 46]. Thus, some of the failure of trichiasis surgery can be attributed to continued or bacterial reinfection, either *C. trachomatis* or other species, and some to genetics. This is echoed in other studies that also show a genetic component to susceptibility of scarring [47, 48].

In a 4-year prospective study [49], trichiasis cumulative recurrence rates at 6 months, 1 year, and 4 years were 32%, 40%, and 41% with significant variation between surgeons in recurrence. The authors and others [49, 50] suggest that early surgery failures were related to surgical factors, such as technique and quality, whereas late failures reflected an ongoing scarring process [51]. Quality assurance was thus suggested to monitor surgery outcomes by surgeon [52]. Because the overall trichiasis recurrence rate was lower than is normally reported for trichiasis surgery in the prospective study, which utilized the posterior tarsal rotation procedure (PLTR), the authors also suggested an evaluation of the WHO recommended BLTR procedure versus the PLTR procedure [49]. Another study [36] in Ethiopia found that recurrence rates increased by surgical variables: longer incisions, their respective placement, and tightness of sutures. The authors also recommend monitoring and supervision, as well as regular retraining of surgeons. 

A randomized controlled trial [53] was undertaken in Ethiopia to determine if absorbable sutures, rather than the standard silk sutures used in surgery, would decrease the risk of recurrence. Although no evidence supported that absorbable sutures reduce the risk of recurrence, they do avoid the common postsurgical complication resulting from patients with silk sutures who do not return for their follow-up 7–10 days after surgery to remove their sutures. This leads to major trauma to the cornea. Even in this study, as many as 2.6% patients with silk sutures did not have them removed until 3 months after surgery. Absorbable sutures do not require removal, so patients' follow-up can be delayed 3–6 months. Since recurrence normally occurs within 6 months, patients needing additional surgery are more easily identified [53]. 

Another common issue with trichiasis surgery are barriers to obtaining the surgery which lead to a decrease in surgical coverage. In a population-based survey in Sokoto State, Nigeria, surgical coverage was very low with rates ranging between 9.5% and 12.5%, even though the government has provided free surgeries since 2003 [54]. The lack of physicians has already been discussed, but there are many other individual barriers that prevent the patient from attending and receiving the surgery. A study [55] of 17 surgical outreach campaigns in Amhara Region, Ethiopia, interviewed 2,591 patients who had previously unoperated trichiasis. Lack of time (45.3%), financial constraints (42.9%), and lack of escort (35.5% in females, 19.6% in males) were the main barriers to surgical uptake. Women were more likely to be afraid of the procedure (7.7% versus 3.2%), be less aware of accessibility of services (4.5% versus 3.2%), and be less likely to have been offered surgery (OR = 0.70) [55].

More than 900,000 surgical treatments have been done worldwide to date, but there needs to be an increase in surgical delivery because the number of surgeries performed does not address the need [2]. Approximately 166,000 surgeries are carried out each year. Thus, major scale-up is needed to fulfill GET 2020 goals [56], especially considering that it will take another 28 years to address the current global TT backlog if surgical productivity does not increase [37]. Taking into account that only 18–66% of patients in Ethiopia agree to surgery, even when it is offered free of charge with free transportation, programs should consider offering an escort for the elderly or bilaterally blind patients, in addition to the patients' other indirect costs and time [33].

## 6. Antibiotic Treatment for Acute Infection

Azithromycin remains the antibiotic of choice for trachoma control, in part because one annual dose over several years, depending on baseline prevalence, appears to eliminate infection. Also, it is much easier to administer orally than the previous unsupervised use of tetracycline ointment for 4–6 weeks. Because reinfection occurs rapidly in endemic regions after treatment, the practice is to treat entire districts annually when the trachoma frequency is greater than 10% in children 1–9 years old. In 1998, Pfizer Inc. and the Edna McConnell Clark Foundation founded the International Trachoma Initiative (ITI). Since 1999, the ITI has coordinated the donation of Zithromax (azithromycin) by Pfizer. To date, Pfizer has donated 225 million treatments (http://www.trachoma.org/), and more than 250 million people have been treated with antibiotics [2].

A side benefit of treating large numbers of people living in fairly close proximity to each other is that, as a whole, the communities show a reduction in other diseases. For example, compared to untreated children, there was a reduction in fever, diarrhea, and vomiting episodes in The Gambia [57], a short-term reduction of diarrhea in Tanzania [58], reduced impetigo in Nepal [59], reduced childhood mortality in Ethiopia [60, 61], and a short-term reduction in the risk of acute lower respiratory infection [62].

Considerable progress has been reported in achieving trachoma elimination [4, 63, 64] or in reducing endemic levels to non-blinding trachoma [65, 66] with azithromycin. However, in a review of randomized trials in which azithromycin was compared to the use of controls, Evans and Solomon [67] concluded that there was considerable variation between trials with unreliable estimates of overall treatment effectiveness. It was difficult to estimate the size of the treatment effect, although they calculated that it was likely to be approximately a 20% relative risk reduction. This may seem a small reduction, but it is very worthwhile. In the randomized trials that compared oral to topical antibiotics, there was no consistent evidence of one being more effective than the other. Among those trials that dealt with the effectiveness of community-based treatment, in which azithromycin was compared to either no or delayed treatment, again, the quality of the evidence was variable. However, one trial produced high-quality evidence that community-based treatment resulted in a reduced prevalence of active trachoma, and infection one year after a single dose. There was also some evidence that oral azithromycin was more effective than tetracycline ointment. The authors concluded that antibiotics reduce the risk and prevalence of active trachoma and ocular chlamydial infection in communities with people infected with* C. trachomatis*, but the size of the treatment in individuals remains uncertain. 

Because *C. trachomatis* infection has been shown to reemerge in communities that have been mass treated [68, 69], it is necessary to undertake repeated rounds of azithromycin treatment to obtain prevalence ratios of less than 5%. The WHO recommends that at least 3 annual treatments should be administered with 80% coverage in communities with a TF prevalence >10% in children 1–9 years, and an impact survey should then determine if antibiotics should be discontinued if prevalence of <5% is achieved [70]. West et al. [69] studied 71 communities in Tanzania, which had received annual mass treatment for 3–7 years. They concluded that for communities which had an initial trachoma level of 50%, annual treatment for more than 7 years may be needed to reach a prevalence of <5%. In a very recent community-randomized trial with the participation of 16 communities in Tanzania, Yohannan et al. [71] hypothesized that if the initial prevalence in a community was 10–20%, then less than 3 rounds of treatment were needed to decrease the prevalence to <5% and, where the estimated prevalence was <5%, the community could stop treatment with antibiotics without reemergence of endemic infection. All communities, regardless if they had initial low prevalence rates, still needed at least 3 rounds of antibiotics. The importance of these findings is that they further support the treatment of an entire, wide geographic area for the WHO recommended 3 rounds, even when some communities are initially lesser endemic.

It has also been concluded that infants under the age of 6 months, who are typically excluded from mass azithromycin treatments, are not a source of reemergent infection [71]. However, because it is still believed that the children with the initially highest bacterial load may be the leading source of reinfection [68], another study [72] tested if 2-day dosing in children with severe trachoma in Tanzania was more effective than 1-day dosing in preventing reinfection after mass treatment. Reduction of infection was significant in both groups with the 2-day dosing group at 96% and the 1-day dosing at 80%. It was generally found that the 2-day dosing group had fewer high-risk children with infection after 6 weeks, which could therefore further reduce the reinfection of the community. However, mass treatment strategies would have to grade trachoma to determine the number of days dosing, which is currently not done. While this study did not consider drug resistance to be a risk, Gebre et al. [73] advocated for less dosage to prevent resistance in children. A random sample of children aged 0–9 years in 12 Ethiopian communities were given annual and twice-annual antibiotics treatments. After 42 months, both the annual and twice-annual groups had similar reinfection rates, suggesting that there may not be significant impact in conducting 2 annual treatments [73]. 

Another potential source of reinfection may be individuals from a community who do not participate in mass treatment. There are a variety of risk factors to consider regarding why individuals are absent from mass treatment. One study [74] looked at the nonparticipation of children in 2 treatment rounds in Tanzania and concluded their guardian risk factors included being of a younger age, perceiving their household health to be excellent at the time of mass treatment, and having less social reliance on the community at large. Household risk factors included family health problems that prevented members from going to the treatment and multiple young children. It was generally difficult to bring all household members to the mass treatment. Many believed that the household and children's needs outweighed the value of the antibiotic treatment. It was suggested that such at-risk households should be targeted by social mobilization programs in the communities. Program risk factors included poor visibility, accessibility, and organization. The main issue was if individuals did not know or recognize their community treatment assistants (CTAs), who are responsible for ensuring community uptake of the mass treatments. CTAs are expected to go door-to-door to households that do not participate in mass treatments, but the study found that this was difficult if they lived more than an hour away from the household. It was recommended to increase the number of distribution days and the number of CTAs to ensure better coverage. While this study cited distance from the CTA as a risk, another study [75] found that the most-difficult-to-reach children were actually less infected in 12 communities in Ethiopia. Provided that 80% of the community members were treated, the authors concluded that it was not necessary to put in the extra time and expense to find absent community members, when a significant increase in the rate of infection was unlikely. Reinfection risk factors for an individual after 3 mass treatments in Ethiopia [76] were ocular discharge (which may be a result of infection), absence at previous mass treatment, living with an infected sibling, living with absentee household members (who might have been traveling at the time of mass treatment), and living in a large community. Bearing in mind that clinical symptoms continue for months after antibiotics clear the initial infection, programs could maximize mass treatment coverage and face washing, provide more prolonged mass treatment to large communities, and distribute antibiotics to children with active trachoma and their siblings after repeated treatments. Research clearly shows the need to reconsider mass treatment approaches and their management on a local community basis to help decrease reinfection rates.

## 7. Face Washing and Environmental Improvement

Because trachoma disappeared from most of the USA and Western Europe before antibiotics were discovered, it was reasoned that trachoma is eliminated by good personal hygiene practices within a sanitary environment. In lesser-developed communities, this requires not only behavioral changes and health education, but also environmental development to improve sanitation and ensure a clean and continuous source of water that is used independently for both hygiene and nonhygiene purposes. The SAFE guidelines incorporate this in the face washing (“F”) and the environmental improvements (“E”) components. The challenge is that trachoma control is primarily managed by/in the public health sector, and there is an overall lack of understanding of behavioral changes and environmental improvements. Therefore, it is necessary to further engage the developmental sector due to their experience working with “F” and “E”—in particular, key potential partners such as the Water Supply and Sanitation Council in Geneva and their Water, Sanitation and Hygiene (WASH) Initiative with UNICEF [77]. 

 Sanitation and environmental improvements can be difficult, even in successful trachoma programs. Nepal, slated to eliminate trachoma by 2014, is one such example [78]. In 1981, the national blindness survey reported that 0.84% of the population was blind; the rate of blindness was 0.34% in 2012, thanks in part to the success of the trachoma program. Approximately 6.0% of the population had trachoma in 1981; today, it is only 0.4%. However, the major barrier to elimination is improved sanitation and hygiene in a country where only 31% of the population has access to these improvements and only 48% is literate, making health education difficult [78]. The trachoma program in Nepal thus underlies the importance of  “F” and “E.” Yet, there is little evidence available of the effectiveness of sanitation and hygiene improvements [79]. 

### 7.1. Face Washing—A Varied Effect

A recent review [80] of clinical trials comparing face washing with no treatment and face washing with antibiotics against antibiotics alone concluded there was some evidence that face washing with topical tetracycline was beneficial, but the evidence generally did not support face washing alone or in combination with antibiotics in reducing active trachoma.

Although a dirty face is considered the pathway by which infection is spread in children with ocular and nasal discharge [81], there is still no standard regarding what constitutes a clean face [82]. A clean face could be defined as an absence of ocular and dry nasal discharge, but that is not a good predictor of whether a face has been washed. 

A very recent study [83] in Ethiopia found that an unclean face, the presence of flies on a face, and the usage of soap were independently associated with active trachoma, but the most important finding for children was that if they were from illiterate households, they were 5 times more likely to have trachoma. Another [84] study in Ethiopia reported that dirty faces and not going to school were significant independent risk factors for children aged 1–9 years. Ocular and nasal discharge in Sudan [85] and flies on a face and a dirty face in Nigeria and Mali [86, 87] were independent risk factors. Flies on a face and nasal discharge were found to be associated with trachoma in Niger [88], but the only significant risk factor was that (rather counterintuitively) the risk of infection increased when the household head had more years of formal education. In Malawi [89], a dirty face was found to be significantly associated with trachoma for univariate analysis, but was not significant for multivariate analysis.

### 7.2. Environmental Improvements—Likewise, a Varied Effect

Evidence in support of environmental improvements is likewise inconsistent and varied. A study [90] in Niger treated all children with tetracycline ointment at baseline, one year, and 2 years following baseline, while randomizing which villages had wells dug that provided clean water. They found no significant differences in the endemic communities, indicating that providing clean water is insufficient to eliminate trachoma [90]. Another study [89] in Niger did not find that latrine use was a factor. Other studies to define the effectiveness of latrine use or of shared versus private latrines in preventing trachoma in rural Tanzania found that latrine use in general decreased the risk of trachoma [91], but that there was no difference in risk between those who shared and those who had their own latrines [92]. Access to a latrine was a protector in Sudan [85], but it was not a protector in one study in Ethiopia [84]. Other studies [83, 93] in Ethiopia produced more inconsistent results. One study [81] found that the frequency of latrine usage was independently associated with trachoma, while another study [93] tested the effect of latrines after mass treatment and found no difference in prevalence with latrine construction only. The latter study further indicates that the implementation of a stand-alone SAFE component (i.e., “E”) is not adequate for meeting program goals.

Other studies on environmental improvements reported that the presence of animal dung in the compound of the household in Nigeria was an independent risk factor [86], and the disposal of waste more than 20 meters away from the household was a protector in Sudan [85]. In Sudan, water accessibility was a possible risk factor [85], whereas it was found to be independently associated with trachoma in Ethiopia [83]. Interestingly, a study [94] in Cameroon found that the lack of a local water source was not linked to the lack of individual or community wealth (to dig wells), but it was due more to lack of social solidarity. The authors recommended that social solidarity should be added to training guides to change behaviors away from waiting for external assistance and dig their own wells. However, individual and community wealth status were considered factors in other studies [83, 85, 87].

 Rabiu et al. [95] performed the first systematic review of 6 different trials in Niger, Ethiopia, The Gambia, Mali, and Tanzania on environmental intervention impact, including hygiene measures related to fly control (insecticides and/or latrine provision), water provision, and education. Conflicting results made it difficult to assess environmental change and impact on active trachoma. Of the 3 insecticide trials reviewed, 2 demonstrated evidence that insecticides may reduce active trachoma, but this was not found in the third trial. Latrine provision did not produce significant results in either of the 2 trials. Health education more successful than behavioral changes in one of the 2 related studies. The authors stressed the need for randomized controlled trials to provide evidence-based results of each environmental intervention and long-term behavioral changes of the community. 

Changing cultural and social behaviors of the community is a major challenge to sanitation and environmental improvements and a barrier to the efforts of health education and increasing community awareness of trachoma. As already discussed, in Cameroon [94], it was discovered that the communities had the means to dig wells and have water easily accessible, but it was long-accepted that the women had to walk far to carry water back to their households. Social norms can indeed inhibit the success of a trachoma program, especially when they are evident at all levels of the community, including in health settings. A baseline Knowledge, Attitudes, and Practices survey was conducted in 2010-2011 in the indigenous Northern Territory of Australia [96]. Responses in regard to the ongoing trachoma program were received from 72 staff members from schools, community workplace, and clinic settings. One-fifth of clinic staff and 29% of school staff were unaware that their community was endemic for trachoma. One-third of school staff and 38% of clinic staff considered children's dirty faces to be normal, which demonstrates the need for more education and awareness on the health risks of unclean faces and the need to persuade the community that a child's dirty face is not acceptable [96]. 

In Mali, innovative and cost-effective solutions have been sought to reach more people with health education and community awareness of trachoma. Since 2008, radio messages about trachoma have been broadcasted [97]. A 2011 survey on the impact of the radio as a conduit for trachoma health education was conducted with the participation of 391 adults and 687 kids in the Kayes and Segou Regions. Most were aware of trachoma, its causes and impact, and how to prevent infection with approximately 60% having heard of it through a radio message. Two-thirds reported that the children washed their face at least twice daily, and 94% said that they used latrines for the disposal of feces. Yet, there was no significant difference in facial cleanliness of children between those whose caretakers heard the radio messages and those who did not, and the authors concluded that it was still necessary to make sanitation, hygiene, and environmental improvement messages clearer [97]. One potential solution that was an important finding resulting from the previously mentioned study on risk factors in Mali [87] was that the presence of a women's association in the 203 participating villages was significantly, negatively associated with trachoma (OR: 0.55; 95% CI 0.36–0.84), which led to the authors' recommendation that the women's associations be the communications network for a health communication directed at changing behaviors.

Although results are inconclusive on the benefits of the “F” and “E” components in reducing active trachoma, the environmental interventions are nevertheless crucial to the overall health and hygiene of the community. There are examples of recently industrialized regions that suggest that improvements in development and health programs have had a significant impact on trachoma. One such example [98] is Sichuan Province, China, which began trachoma control in the 1960s, when 55% of China's urban population and up to 90% of its rural population had trachoma. Chen et al. [98] compared the 1987 and 2006 National Sample Survey on Disabilities, in which 125,000 people participated in Sichuan. In 1987, blinding trachoma was the second leading cause of the 8 visual impairments surveyed, found in 172.9 people per 100,000 population. In 2006, trachoma was the eighth cause of visual impairment and the only reduced cause of blindness (58 people per 100,000 populations). Only people over 40 years had blinding trachoma.

## 8. The Overall Effectiveness of SAFE

A large number of recent studies have provided evidence that implementation of all the aspects of the SAFE guidelines leads to reduction of trachoma and trachoma-associated blindness [4, 60, 63–66, 99–105]. In 14 villages in The Gambia, a single mass dose of azithromycin was given at baseline (83% coverage). All families had access to clean water, and latrines were installed for all households. The prevalence of TF was 15.4%, and *C. trachomatis* was present in 9.7% of children 1–9 years old. After 5 years, the prevalence of infection dropped to 0% in 12 villages. In the other 2 villages, treatment was initially followed by increased infection, which was attributed to widespread contact with untreated communities and then fell to 0% prevalence. By the end of the study [99], the prevalence of trachoma was 0.6% for the entire population. However, in communities that had higher initial prevalence of disease, a single mass treatment was insufficient to lower trachoma prevalence substantially [4, 60, 64, 100–107].

In contrast, an attempt to implement the SAFE strategy in Australia was clearly met with less than complete success [31]. Environmental interventions included road sealing to cut down on dust; planting native plants, trees, and grass for additional dust control; replacement of poorly built and/or maintained houses; biweekly trash collection; heating/cooling systems repair; upgrades in sewer lines; and installing rainwater tanks. Schools instituted health education on personal hygiene. After extensive assessment, azithromycin was distributed to 2 villages. There was 73% coverage in the village with environmental improvements (TF + TI = 48%). The control village (TF + TI = 51%), also received mass azithromycin with 55% coverage. Three months after mass treatment, the prevalence (TF + TI) was reduced to 21.2% in the improved village and 24.2% in the control village. At one year, the environmentally improved village prevalence remained stable, and the control village had 30.0% prevalence. Both reductions from baseline were significant. It remains to be seen whether better azithromycin coverage would alter the minimal effectiveness of environmental improvements. A small sample survey [108] of 14 health professionals directly involved in trachoma programs in the Northern Territory demonstrated that the challenges of trachoma programming may be due to trachoma being a low health priority, the lack of program leadership and failure of the program structure, and the overall lack of resources and properly trained staff (with high staff turnover reported). More community support was needed to make the program more successful. Part of the issue in Australia is that many believe that trachoma is no longer a problem [109]. The good news is that since 2006, the National Trachoma Surveillance and Reporting Unit (NTSRU) has been collecting annual data, improving grading methods, and increasing screening and treatment coverage. These efforts have been further boosted by the 2009 Indigenous Eye and Ear Health Initiative, which provided a 4-year budget of $16 million to eliminate trachoma; however, for this initiative to be successful, it must be long term [109]. Indeed, as of 2010, NTSRU data from the aboriginal regions where trachoma is endemic revealed that treatment coverage (at 70%) varied widely, there were limited data available on the burden of trichiasis in adults (indicating a need for more screening), and the populations to be screened remained undefined [110]. In general, the screening and treatment strategies in Australia need to be reconsidered to optimize the impact of the trachoma programs and SAFE strategy, especially in low prevalence areas [111].

Gender may be another social issue hindering the success of the SAFE strategy in some areas. Considering that females are at higher risk and more vulnerable to infection, one would think that screening and treatment efforts would be more targeted towards the female population. This is the case in many programs, but the gender gap remains a problem in Oman, which may not successfully eliminate trachoma until coverage is more equal between men and women [40]. In underdeveloped communities of India, as many as one-third of women had trachoma symptoms, but only 40% received any treatment [112].

### 8.1. A Closer Consideration of *Chlamydia Trachomatis* for the “A” Component of SAFE Strategy

In culture, *C. trachomatis* has been demonstrated repeatedly to enter a viable, persistent state, in which the organism cannot be cultivated but is in a long-term relationship with the host. Certain cytokines such as interferon-*γ* (IFN-*γ*), antibiotics treatment, and withholding of some nutrients cause the parasite to become persistent. In the persistent state, the bacterium is refractory to antibiotic treatment and has very little metabolic activity [113, 114]. Persistence caused by azithromycin can be reversed by adding tryptophan [113–116]. When the persistent state is induced in cells in culture by tryptophan starvation, the persistent state can be reversed by supplying tryptophan [113]. However, with prolonged starvation, the bacterium dies. 

Infection also causes IFN-*γ* to be produced, resulting in a persistent state by inducing an enzyme, indoleamine 2,3-dioxygenase, that breaks down tryptophan [117, 118]. This appears to be the major innate immune mechanism in controlling *C. trachomatis* growth in cells [119]. The morphological changes that occur with INF-*γ* induction of persistence are identical to those with azithromycin [120]. As with azithromycin and tryptophan-starvation-induced persistence, the induction of tryptophan depletion and persistence by INF-*γ* can be reversed by the provision of tryptophan. This results in a reversion of the morphology, and the organism proceeds with its normal life cycle. The innate immune response induces a state in which the bacterium is immune to azithromycin. Moulder [113] investigated the time necessary for persistence to be irreversible by tryptophan. When cells were treated with IFN-*γ* and held in the persistent state for 1 day, the infection inhibition was reversed completely by tryptophan. After 2 days, tryptophan was partially able to reverse infection. After 3 days, reversal was hardly detectable, and it did not occur at all for longer periods. This strongly suggests that patients must be given enough doses of azithromycin so that the bacterium cannot survive in persistence. One dose of azithromycin, with its half-life of 11–14 hours [121], may not be sufficient to induce irreversible persistence in a small subpopulation of individuals, who may serve as a reservoir for new infection.

Singla [117] has suggested that azithromycin be accompanied by tryptophan in treatment. His theory is that persistence can be avoided or reversed by tryptophan, making the bacterium vulnerable to the antibiotic. However, since one dose of azithromycin has a half-life of only 11–14 hours, it may require two or more sequential doses. After treatment, it takes approximately 9 hours for the bacterium to recover its active state. That is unlikely to be sufficient time for the prolonged period necessary to kill the bacterium. Alternatively, because azithromycin kills by holding the bacterium in a persistent state, it may be that the combination of tryptophan reversal of persistence needs to be coupled with another antibiotic, one that kills directly. All of these aspects need to be further investigated. Due to scarring from inflammation, it is also reasonable to ask whether anti-inflammatories might help to prevent the scarring from trachoma.

Finally, the study of Burton et al. [18] in Tanzania reiterates that in lesser endemic regions, other pathogens may be responsible for the TF clinical signs. The presence of bacterial pathogens (most commonly *S. pneumoniae* and *H. influenza*) was associated with TF (odds ratio: 4.7), suggesting along with other studies, that in individuals who have previously had TF, a subsequent episode in which conjunctival follicles are observed could be due to other pathogens.

In summary, although the implementation of the SAFE guidelines has been reported to reduce and/or eliminate blinding trachoma from regions and even entire countries, there remain the troublingly high rate of surgical failures, the puzzling continued inflammation in the absence of detectable infection that contributes to progression of the disease process and surgical failure, the less-than-high rate of treatment effectiveness of azithromycin, repeated reinfection even under conditions of high coverage, and the minimal impact of environmental improvements. Trachoma can be eliminated if all components of SAFE are in place for at least 5 years, but even after elimination in children, surgery may be needed for some time to clear the adult backlog [77]. Scale-up is needed to fulfill the GET 2020 goals.

### 8.2. SAFE Strategy and Integration with Neglected Tropical Diseases—A Scale-Up Solution?

An emerging solution to scale-up and to run more efficient and effective programs is neglected tropical diseases (NTDs) integration. Dembéle et al. [122] highlighted the national NTD control program in Mali, which since 2007 has targeted the 5 major NTDs endemic in Mali: lymphatic filariaiss, onchocerciasis, schistosomiasis, soil-transmitted helminthiasis, and trachoma. The program has been particularly successful in scaling up mass treatment. Since 2009, geographic coverage has been at 100%, and trachoma program coverage has been from 76 to 97%. This success is due to integrated drug delivery in the primary health care system, which now treats 10 million people each year. Considering that there are at least 1.9 billion worldwide needing treatment for at least one NTD, and 33% of them need drugs for 3 or more [123], integrated drug therapy should be further explored for trachoma. There are logistical challenges with NTD integration, such as the need for monitoring of drug efficacy [123], ensuring all drug orders are placed in a timely fashion to guarantee distribution, maintaining an up-to-date inventory of the drugs, and insufficient funding for monitoring and surveillance [122]. Indeed, funding is the main issue, especially considering that other public health programs such as malaria, tuberculosis, or HIV/AIDs give more financial incentives to the primary care health system. About half of the US$1.5-2 billion NTD budget is needed, but if 10% of what donors give to malaria, tuberculosis, or HIV/AIDS were allocated to NTDs, the main NTDs, including trachoma, could be eliminated [124]. The WHO agrees that scale-up of trachoma programming may be easier through NTD integration, but improving and increasing surgery requires immediate attention [2]. NTD integration appears to be most beneficial to mass treatment of the SAFE strategy [125], but integration trachoma and NTD is a subject of its own for another review or investigation.

## 9. Discussion

Considerable progress has been made since the inception of GET 2020 in lowering the prevalence of trachoma worldwide. However, more emphasis on reducing repeated infection cycles to prevent corneal scarring and ultimately blindness is required, in addition to improvement in surgical quality and increasing surgical productivity. Fine tuning of mass azithromycin administration protocols in communities could be helpful, and the relationship between environmental factors and the reduction of trachoma prevalence needs further investigation. 

A review [126] of recent research in the prevention, diagnosis, and treatment of trachoma concluded that the SAFE strategy's mass treatment with antibiotics is the mainstay of trachoma treatment and is why community-targeted strategies are key to program success. Future research could focus on optimizing mass treatment by investigating integrated monitoring and treatment strategies at a community level. While the review covers a narrower timeframe and scope of literature, the most important conclusion reflects an overall finding of this current review—that trachoma elimination is possible even in hyperendemic communities [126].

This current review included a considerable more number of studies related to the SAFE strategy and/or its specific components spanning 15 years from 1998 through February 2013. However, it is necessary to reiterate that this review does not include every publication from that time frame, which may be a potential limitation and lead to bias. The focus was on original findings of the literature as they related to the overall effectiveness of the SAFE strategy. Much more literature is currently available on the genetic make-up, predictor models, and pathogenesis of trachoma, which may be a further indication of risk factors and prevention methods. Another limitation of this review is that it included all types of studies and levels of evidence. Some of the findings from the studies included in this study may be weak based on study design and/or based on very limited sample sizes that might lead to bias, and this review does not evaluate the studies based on their strength and high-level evidence. A systematic review on the SAFE strategy would be needed for this type of analysis. Also, randomized controlled trials, which are considered the gold standard of evidence, are very limited in trachoma research. What are needed are more clinical trials, not only in hyperendemic communities, but also in communities with low prevalence, with research questions based on the ongoing puzzlements of trachoma control and prevention to improve screening and diagnosis of infection, to introduce better techniques to improve surgical outcome, to increase the effect of mass treatment of azithromycin (particularly on recurrence of infection), and to seek to understand and better implement hygiene and environmental improvements. At the same time, the local surveys, cross-sectional population studies, and risk factors assessments reviewed still bear merit considering that the focus on trachoma control and prevention strategy is increasingly aimed at the local community.

## 10. Conclusions

The global elimination of trachoma is approaching reality. This current review further revealed that it is generally agreed that scale-up is needed for all SAFE components, but more evidence is needed to show that scale-up by NTD integration will specifically improve trachoma control. More research is also needed in understanding the effect and impact of environmental improvements on prevention. Finally, as more endemic countries/regions become involved in trachoma control thanks to the global awareness raised by GET 2020, more research should be generated from communities outside of Africa and Asia.

## Figures and Tables

**Table 1:** World Health Organization simplified classification of trachoma infection.

Grade	Description
Follicular trachoma (TF)	The presence of 5 or more follicles (of at least 0.5 mm) in the upper tarsal conjunctiva
Inflammatory trachoma (TI)	Pronounced inflammatory thickening of the tarsal conjunctiva that obscures more than half of the deep normal tarsal vessels
Trachomatous scarring (TS)	The presence of scarring in the tarsal conjunctiva
Trichiasis (TT)	At least one lash touches the eyeball
Corneal opacity (CO)	The presence of easily visible corneal opacity which obscures at least some of the pupil

## References

[1] Smith JL, Haddad D, Polack S (2011). Mapping the global distribution of Trachoma: why an updated Atlas is needed. *PLoS Neglected Tropical Diseases*.

[2] World Health Organization (2012). Global WHO alliance for the elimination of blinding trachoma by 2020. *Weekly Epidemiological Record*.

[3] Thylefors B, Negrel AD, Pararajasegaram R, Dadzie KY (1995). Global data on blindness. *Bulletin of the World Health Organization*.

[4] World Health Organization Report of the twelfth meeting of the WHO alliance for the global elimination of blinding trachoma.

[5] Gambhir M, Basáñez MG, Blake IM, Grassly NC (2010). Modelling trachoma for control programmes. *Advances in Experimental Medicine and Biology*.

[6] World Health Organization http://gamapserver.who.int/mapLibrary/Files/Maps/Global_trachoma_2010.png.

[7] The Trachoma Atlas http://www.trachomaatlas.org/.

[8] Burton MJ, Bailey RL, Jeffries D, Mabey DCW, Holland MJ (2004). Cytokine and fibrogenic gene expression in the conjunctivas of subjects from a Gambian community where trachoma is endemic. *Infection and Immunity*.

[9] Skwor T, Kandel RP, Basravi S, Khan A, Sharma B, Dean D (2010). Characterization of humoral immune responses to chlamydial HSP60, CPAF, and CT795 in inflammatory and severe trachoma. *Investigative Ophthalmology & Visual Science*.

[10] Kari L, Whitmire WM, Crane DD (2009). Chlamydia trachomatis native major outer membrane protein induces partial protection in nonhuman primates: implication for a trachoma transmission- blocking vaccine. *Journal of Immunology*.

[11] West SK (2004). Trachoma: new assault on an ancient disease. *Progress in Retinal and Eye Research*.

[12] Wolle MA, Muñoz BE, Mkocha H, West SK (2009). Constant ocular infection with chlamydia trachomatis predicts risk of scarring in children in Tanzania. *Ophthalmology*.

[13] Gouda H, Powles J, Barendregt J, Emerson P, Ngondi J (2012). The burden of trachoma in South Sudan: assessing the health losses from a condition of graded severity. *PLOS Neglected Tropical Diseases*.

[14] Bailey R, Duong T, Carpenter R, Whittle H, Mabey D (1999). The duration of human ocular Chlamydia trachomatis infection is age dependent. *Epidemiology and Infection*.

[15] Faal N, Bailey RL, Jeffries D (2006). Conjunctival FOXP3 expression in trachoma: do regulatory T cells have a role in human ocular Chlamydia trachomatis infection?. *PLoS Medicine*.

[16] Cevallos V, Whitcher JP, Melese M (2012). Association of conjunctival bacterial infection and female sex in cicatricial trachoma. *Investigative Ophthalmology & Visual Science*.

[17] Gallenga PE, Del Boccio M, Rapinese M, DiIorio A, Toniato E, Martinotti S (2011). Molecular approach by PCR is the best method to detect the presence of Chlamydia trachomatis and to define the true agent of ocular bacterial inflammation. *International Journal of Immunopathology and Pharmacology*.

[18] Burton MJ, Hu VH, Massae P (2011). What is causing active trachoma? The role of non-chlamydial bacterial pathogens in a low prevalence setting. *Investigative Ophthalmology & Visual Science*.

[19] Yang JL, Hong KC, Schachter J (2009). Detection of Chlamydia trachomatis ocular infection in trachoma-endemic communities by rRNA amplification. *Investigative Ophthalmology & Visual Science*.

[20] Keenan JD, Ayele B, Gebre T (2012). Ribomsomal RNA evidence of ocular chlamydia trachomatis infection following 3 annual mass azithromycin distributions in communities with highly prevalent trachoma. *Clinical Infectious Diseases*.

[21] Keenan JD, See CW, Moncada J (2012). Diagnostic test characteristics of tests for ocular chlamydia after mass azithromycin distributions. *Investigative Ophthalmology & Visual Science*.

[22] See CW, Alemayebu W, Melese M (2011). How reliable are tests for trachoma?-a latent class approach. *Investigative Ophthalmology & Visual Science*.

[23] Andreasen AA, Burton MJ, Holland MJ (2008). Chlamydia trachomatis ompA variants in trachoma: what do they tell us?. *PLoS Neglected Tropical Diseases*.

[24] Caldwell HD, Wood H, Crane D (2003). Polymorphisms in Chlamydia trachomatis tryptophan synthase genes differentiate between genital and ocular isolates. *The Journal of Clinical Investigation*.

[25] Wright HR, Turner A, Taylor HR (2008). Trachoma. *The Lancet*.

[26] Mkocha H, Munoz B, West S (2009). Trachoma and ocular Chlamydia trachomatis rates in children in trachoma-endemic communities enrolled for at least three years in the Tanzania National Trachoma Control Programme. *Tanzania Journal of Health Research*.

[27] Michel CE, Roper KG, Divena MA, Lee HH, Taylor HR (2011). Correlation of clinical trachoma in Aboriginal communities. *PLOS Neglected Tropical Diseases*.

[28] Munoz B, Stare D, Mkocha H (2011). Can clinical signs of trachoma be used after multiple rounds of mass antibiotic treatment to indicate infection?. *Investigative Ophthalmology & Visual Science*.

[29] Solomon AW, Peeling RW, Foster A, Mabey DCW (2004). Diagnosis and assessment of trachoma. *Clinical Microbiology Reviews*.

[30] Yayemain D, King JD, Debrah O (2009). Achieving trachoma control in Ghana after implementing the SAFE strategy. *Transactions of the Royal Society of Tropical Medicine and Hygiene*.

[31] Lansingh VC, Mukesh BN, Keeffe JE, Taylor HR (2010). Trachoma control in two Central Australian Aboriginal communities: a case study. *International Ophthalmology*.

[32] Ferreira IS, Bernardes TF, Bonfioli AA (2010). Trichiasis. *Seminars in Ophthalmology*.

[33] Rajak SN, Collin JRO, Burton MJ (2012). Major Review: trachomatous trichiasis and its management in endemic countries. *Survey of Ophthalmology*.

[34] Rajak SN, Habtamu E, Weiss HA (2011). Surgery versus epilation for the treatment of minor trichiasis in Ethiopia: a randomised controlled noninferiority trial. *PLOS Medicine*.

[35] Gower EW, West SK, Harding JC (2013). Trachomatous trichiasis clamp vs standard bilamellar tarsal rotation instrumentation for trichiasis surgery. Results from a randomized clinical trial. *JAMA Ophthalmology*.

[36] Woreta TA, Munoz BE, Gower EW, Alemayehu W, West SK (2009). Effect of trichiasis surgery on visual acuity outcomes in Ethiopia. *Archives of Ophthalmology*.

[37] Burton MJ Global trichiasis scientific meeting report.

[38] Burton MJ, Kinteh F, Jallow O (2005). A randomised controlled trial of azithromycin following surgery for trachomatous trichiasis in the Gambia. *British Journal of Ophthalmology*.

[39] Bowman RJC, Faal H, Myatt M (2002). Longitudinal study of trachomatous trichiasis in the Gambia. *British Journal of Ophthalmology*.

[40] Khandekar RB, Al Harby SS, Vora UP (2012). Lid surgery for trachomatous trichiasis is negatively associated with visual disabilities and visual impairments in Oman. *Eastern Mediterranean Health Journal*.

[41] West SK, West ES, Alemayehu W (2006). Single-dose azithromycin prevents trichiasis recurrence following surgery: randomized trial in Ethiopia. *Archives of Ophthalmology*.

[42] West S, Alemayehu W, Munoz B, Gower EW (2007). Azithromycin prevents recurrence of severe trichiasis following trichiasis surgery: STAR trial. *Ophthalmic Epidemiology*.

[43] Woreta F, Munoz B, Gower E, Alemayehu W, West SK (2012). Three-year outcomes of the surgery for trichiasis, antibiotics to prevent recurrence trial. *Archives of Ophthalmology*.

[44] Burton MJ, Bailey RL, Jeffries D (2010). Conjunctival expression of matrix metalloproteinase and proinflammatory cytokine genes after trichiasis surgery. *Investigative Ophthalmology & Visual Science*.

[45] Conway DJ, Holland MJ, Bailey RL (1997). Scarring trachoma is associated with polymorphism in the tumor necrosis factor alpha (TNF-*α*) gene promoter and with elevated TNF-*α* levels in tear fluid. *Infection and Immunity*.

[46] Natividad A, Hanchard N, Holland MJ (2007). Genetic variation at the TNF locus and the risk of severe sequelae of ocular Chlamydia trachomatis infection in Gambians. *Genes and Immunity*.

[47] Natividad A, Hull J, Luoni G (2009). Innate immunity in ocular Chlamydia trachomatis infection: contribution of IL8 and CSF2 gene variants to risk of trachomatous scarring in Gambians. *BMC Medical Genetics*.

[48] Mozzato-Chamay N, Mahdi OSM, Jallow O, Mabey DCW, Bailey RL, Conway DJ (2000). Polymorphisms in candidate genes and risk of scarring trachoma in a Chlamydia trachomatis-endemic population. *Journal of Infectious Diseases*.

[49] Rajak SN, Makalo P, Sillah A (2010). Trichiasis surgery in the Gambia: a 4-year prospective study. *Investigative Ophthalmology & Visual Science*.

[50] Khandekar R, Thanh TT, Luong VQ (2009). The determinants of trichiasis recurrence differ at one and two years following lid surgery in Vietnam: a community-based intervention study. *Oman Journal of Ophthalmology*.

[51] Gower EW, Merbs SL, Munoz BE (2011). Rates and risk factors for unfavorable outcomes 6 weeks after trichiasis surgery. *Investigative Ophthalmology & Visual Science*.

[52] Buchan JC, Limburg H, Burton MJ (2011). Quality assurance in trichiasis surgery: a methodology. *British Journal of Ophthalmology*.

[53] Rajak SN, Habtamu E, Weiss HA (2011). Absorbable versus silk sutures for surgical treatment of trachomatous trichiasis in Ethiopia: a randomized controlled trial. *PLOS Medicine*.

[54] Muhammad N (2012). Trichiasis surgical coverage in three local government areas of Sokoto state, Nigeria. *Annals of African Medicine*.

[55] Rajak SN, Habtamu E, Weiss HA (2012). Why do people not attend for treatment for trachomatous trichiasis in Ethiopia? A study of barriers to surgery. *PLOS Neglected Tropical Diseases*.

[56] Kello AB (2012). *Scaling up the Coverage of Quality Trichiasis Surgery*.

[57] Sadiq ST, Glasgow KW, Drakeley CJ (1995). Effects of azithromycin on malariometric indices in The Gambia. *The Lancet*.

[58] Coles CL, Seidman JC, Levens J, Mkocha H, Munoz B, West S (2011). Association of mass treatment with azithromycin in trachoma-endemic communities with short-term reduced risk of diarrhea in young children. *The American Journal of Tropical Medicine and Hygiene*.

[59] Fry AM, Jha HC, Lietman TM (2002). Adverse and beneficial secondary effects of mass treatment with azithromycin to eliminate blindness due to trachoma in Nepal. *Clinical Infectious Diseases*.

[60] Porco TC, Gebre T, Ayele B (2009). Effect of mass distribution of azithromycin for trachoma control on overall mortality in Ethiopian children: a randomized trial. *Journal of the American Medical Association*.

[61] Keenan JD, Ayele B, Gebre T (2011). Childhood mortality in a cohort treated with mass azithromycin for trachoma. *Clinical Infectious Diseases*.

[62] Coles CL, Levens J, Seidman JC (2011). Mass distribution of azithromycin for trachoma control is associated with short-term reduction in risk of acute lower respiratory infection in young children. *The Pediatric Infectious Disease Journal*.

[63] Amza A, Goldschmidt P, Einterz E (2010). Elimination of active trachoma after two topical mass treatments with azithromycin 1.5% eye drops. *PLoS Neglected Tropical Diseases*.

[64] Biebesheimer JB, House J, Hong KC (2009). Complete elimination of infectious trachoma from severely affected communities after six biannual mass azithromycin distributions. *Ophthalmology*.

[65] Solomon AW, Holland MJ, Alexander NDE (2004). Mass treatment with single-dose azithromycin for trachoma. *The New England Journal of Medicine*.

[66] Chidambaram JD, Alemayehu W, Melese M (2006). Effect of a single mass antibiotic distribution on the prevalence of infectious trachoma. *Journal of the American Medical Association*.

[67] Evans JR, Solomon AW (2011). Antibiotics for trachoma. *Cochrane Database of Systematic Reviews*.

[68] West SK, Munoz B, Mkocha H (2005). Infection with Chlamydia trachomatis after mass treatment of a trachoma hyperendemic community in Tanzania: a longitudinal study. *The Lancet*.

[69] West SK, Munoz B, Mkocha H, Gaydos CA, Quinn TC (2011). Number of years of annual mass treatment with azithromycin needed to control trachoma in hyper-endemic communities in Tanzania. *Journal of Infectious Diseases*.

[70] Solomon A, Zondervan M, Kuper H, Buchan JC, Mabey DCW, Foster A (2006). *Trachoma Control: A Guide for Programme Managers*.

[71] Yohannan J, Munoz B, Mkocha H (2013). Can we stop mass drug administration prior to 3 annual rounds in communities with low prevalence of trachoma? PRET Ziada Trial Results. *JAMA Ophthalmology*.

[72] Campbell JP, Mkocha H, Munoz B, West SK (2012). Two-day dosing versus one-day dosing of Azithromycin in children with severe trachoma in Tanzania. *Ophthalmic Epidemiology*.

[73] Gebre T, Ayele B, Zerihun M (2012). Comparison of annual versus twice-yearly mass azithromycin treatment for hyperendemic trachoma in Ethiopia: a cluster randomised trial. *The Lancet*.

[74] Ssemanda EN, Levens J, MKocha H (2012). Azithromycin mass treatment for trachoma control risk factors for non-participation of children in two treatment rounds. *PLOS Neglected Tropical Diseases*.

[75] Keenan JD, Moncada J, Gebre T (2012). Chlamydial infection during trachoma monitoring: are the most difficult to reach children more likely to be infected?. *Tropical Medicine & International Health*.

[76] Ayele B, Gebre T, Moncada J (2011). Risk factors for ocular chlamydia after three mass azithromycin distributions. *PLOS Neglected Tropical Diseases*.

[77] Bush S (2012). *Can Trachoma be Eliminated in the Next Decade: What are the Challenges?*.

[78] Yee A (2012). Nepal sees end in sight for trachoma. *The Lancet*.

[79] Rabiu M, Alhassan MB, Ejere HOD, Evans JR (2012). *Environmental Sanitary Interventions for Preventing Active Trachoma (Review)*.

[80] Ejere HOD, Alhassan MB, Rabiu M (2012). *Face Washing Promotion for Preventing Active Trachoma (Review)*.

[81] Taylor HR (2008). *Trachoma: A Blinding Scourge from the Bronze Age to the Twenty-First Century*.

[82] King JD, Ngondi J, Kasten J (2011). Randomised trial of face-washing to develop a standard definition of a clean face for monitoring trachoma control programmes. *Transactions of the Royal Society of Tropical Medicine and Hygiene*.

[83] Ketema K, Tirunen M, Woldeyohannes D, Muluye D (2012). Active trachoma and associated risk factors among children in Baso Liben District of East Gojjam, Ethiopia. *BMC Public Health*.

[84] Roba AA, Patel D, Zondervan M (2013). Risk of trachoma in a SAFE intervention area. *International Ophthalmology*.

[85] Edwards T, Smith J, Sturrock HJW (2012). Prevalence of trachoma in Unity State, South Sudan: results from a large-scale population-based survey and potential implications for further surveys. *PLOS Neglected Tropical Diseases*.

[86] Mypyet C, Lass BD, Yanaya HB, Solomon AW (2012). Prevalence of and risk factors for trachoma in Kano State, Nigeria. *PLoS One*.

[87] Hägi M, Schémann JF, Mauny F (2010). Active trachoma among children in Mali: clustering and environmental risk factors. *PLOS Neglected Tropical Diseases*.

[88] Amza A, Kadri B, Nassirou B (2012). Community risk factors for ocular chlamydia infection in Niger: pre-treatment results from a cluster-randomized trachoma trial. *PLOS Neglected Tropical Diseases*.

[89] Kalua K, Chirwa T, Kalilani L, Abbenyi S, Mukaka M, Bailey R (2010). Prevalence and risk factors for trachoma in central and southern Malawi. *PLoS One*.

[90] Abdou A, Munoz BE, Nassirou B (2010). How much is not enough? A community randomized trial of a Water and Health Education programme for Trachoma and Ocular C. trachomatis infection in Niger. *Tropical Medicine and International Health*.

[91] Montgomery MA, Desai MM, Elimelech M (2010). Assessment of latrine use and quality and association with risk of trachoma in rural Tanzania. *Transactions of the Royal Society of Tropical Medicine and Hygiene*.

[92] Montgomery MA, Desai MM, Elimelech M (2010). Comparing the effectiveness of shared versus private latrines in preventing trachoma in Rural Tanzania. *American Journal of Tropical Medicine and Hygiene*.

[93] Stoller NE, Gebre T, Ayele B (2011). Efficacy of latrine promotion on emergence of infection with ocular Chlamydia trachomatis after mass antibiotic treatment: a cluster-randomized trial. *International Health*.

[94] Goldschmidt P, Einterz E, Bates M, Abba F, Chaumeil C, Bensaid P (2012). Contributions to the improvement of living conditions among neglected populations with trachoma. *Tropical Medicine and Health*.

[95] Rabiu M, Alhassan MB, Ejere HOD (2009). *Environmental Sanitary Interventions for Preventing Active Trachoma (Review)*.

[96] Lange FD, Baunach E, McKenzie R, Taylor HR (2012). Trachoma elimination in remote Indigenous Northern Territories communities: baseline health-promotion study. *Australian Journal of Public Health*.

[97] Bamani S, Toubali F, Diarra S (2013). Enhancing community knowledge and health behaviors to eliminate blinding trachoma in Mali using radio messaging as a strategy. *Health Education Research*.

[98] Chen H, Wu X, Wei M (2012). Changes in the prevalence of visual impairment due to blinding trachoma in Sichuan Province, China: a comparative study between 1987–2006. *Ophthalmic Epidemiology*.

[99] Burton MJ, Holland MJ, Makalo P (2010). Profound and sustained reduction in Chlamydia trachomatis in The Gambia: a five-year longitudinal study of trachoma endemic communities. *PLoS Neglected Tropical Diseases*.

[100] Roba AA, Wondimu A, Patel D, Zondervan M (2011). Effects of intervention with the SAFE strategy on trachoma across Ethiopia. *Journal of Epidemiology and Community Health*.

[101] Khandekar R, Mabry R, Al Hadrami K, Sarvanan N (2005). Active trachoma, face washing (F) and environmental improvement (E) in a high-risk population in Oman. *Eastern Mediterranean Health Journal*.

[102] Ngondi J, Matthews F, Reacher M, Baba S, Brayne C, Emerson P (2008). Associations between active trachoma and community intervention with antibiotics, facial cleanliness, and environmental improvement (A,F,E). *PLoS Neglected Tropical Diseases*.

[103] Ngondi J, Gebre T, Shargie EB (2010). Estimation of effects of community intervention with antibiotics, facial cleanliness, and environmental improvement (A,F,E) in five districts of ethiopia hyperendemic for trachoma. *British Journal of Ophthalmology*.

[104] Hagan M, Yayemain D, Ahorsu F, Aboe A (2009). Prevalence of active trachoma two years after control activities. *Ghana Medical Journal*.

[105] Yayemain D, King JD, Debrah O (2009). Achieving trachoma control in Ghana after implementing the SAFE strategy. *Transactions of the Royal Society of Tropical Medicine and Hygiene*.

[106] Ngondi J, Gebre T, Shargie EB (2009). Evaluation of three years of the SAFE strategy (surgery, antibiotics, facial cleanliness and environmental improvement) for trachoma control in five districts of Ethiopia hyperendemic for trachoma. *Transactions of the Royal Society of Tropical Medicine and Hygiene*.

[107] Ngondi J, Gebre T, Shargie EB (2010). Estimation of effects of community intervention with antibiotics, facial cleanliness, and environmental improvement (A,F,E) in five districts of ethiopia hyperendemic for trachoma. *British Journal of Ophthalmology*.

[108] Wright HR, Keeffe JE, Taylor HR (2010). Barriers to the implementation of the safe strategy to combat hyperendemic Trachoma in Australia. *Ophthalmic Epidemiology*.

[109] Taylor HR, Anjou MD (2012). Trachoma in Australia: an update. *Clinical and Experimental Ophthalmology*.

[110] National Trachoma Surveillance and Reporting Unit (2011). *Australian Trachoma Surveillance Report 2010*.

[111] Jones S, Whitehead O, Brian G (2012). Trachoma in North Queensland: an example of poor population health practice. *Clinical and Experimental Ophthalmology*.

[112] Khanduja S, Jhanji V, Sharma N (2012). Trachoma prevalence in women living in rural northern India: rapid assessment findings. *Ophthalmic Epidemiology*.

[113] Moulder JW (1991). Interaction of Chlamydiae and host cells in vitro. *Microbiological Reviews*.

[114] Hammerschlag MR (2002). The intracellular life of Chlamydiae. *Seminars in Pediatric Infectious Diseases*.

[115] Engel JN (1992). Azithromycin-induced block of elementary body formation in Chlamydia trachomatis. *Antimicrobial Agents and Chemotherapy*.

[116] Clark RB, Schatzki PF, Dalton HP (1982). Ultrastructural analysis of the effects of erythromycin on the morphology and developmental cycle of Chlamydia trachomatis HAR-13. *Archives of Microbiology*.

[117] Singla M (2007). Role of Tryptophan supplementation in the treatment of Chlamydia. *Medical Hypotheses*.

[118] Beatty WL, Belanger TA, Desai AA, Morrison RP, Byrne GI (1994). Tryptophan depletion as a mechanism of gamma interferon-mediated chlamydial persistence. *Infection and Immunity*.

[119] Roshick C, Wood H, Caldwell HD, McClarty G (2006). Comparison of gamma interferon-mediated antichlamydial defense mechanisms in human and mouse cells. *Infection and Immunity*.

[120] Beatty WL, Byrne GI, Morrison RP (1993). Morphologic and antigenic characterization of interferon *γ*-mediated persistent Chlamydia trachomatis infection in vitro. *Proceedings of the National Academy of Sciences of the United States of America*.

[121] Lode H (1991). The pharmacokinetics of azithromycin and their clinical significance. *European Journal of Clinical Microbiology & Infectious*.

[122] Dembéle M, Bamani S, Dembélé R (2012). Implementing preventive chemotherapy through and integrated national neglected tropical disease control program in Mali. *PLOS Neglected Tropical Diseases*.

[123] Engels D Update on integrated framework development for preventive chemotherapy interventions.

[124] Fenwick A (2012). The global burden of neglected tropical diseases. *Public Health*.

[125] Haddad D (2012). *The End in Sight: A Global Strateg to Eliminate Blinding Trachoma*.

[126] Bhosai SJ, Bailey RL, Gaynor BD, Leitman TM (2012). Trachoma: an update on prevention, diagnosis, and treatment. *Current Opinion in Ophthalmology*.

